# Rats on Shipboard

**Published:** 1904-12-17

**Authors:** 


### The Hospital.

A Journal of

The Medical Sciences and Hospital Administration.

Vol. XXXVII.—No. 951. DECEMBER 17, 1904.

### Rats on Shipboard.

The accusations which were freely brought against rats, a year or two ago, as being probably among the chief agencies concerned in the conveyance of bubonic plague from country to country, cannot be said to have been completely sustained by evidence; for, although the animals unquestionably suffer and perish from the disease, their instrumentality in its diffusion is less certain. Precautions against this instrumentality are perhaps less insisted upon now than formerly; but, nevertheless, they err, if at all, upon the side of safety; and experiments concerning the best ways of destroying rats on shipboard must always possess an interest for sanitarians. A valuable account of such experiments has just been published by the Local Government Board, in the form of a report to the Board by Dr. Haldane, F.R.S., and Dr. Wade, a report which, although dealing primarily with the question of rats, deals incidentally with the destructive effects of rat killing agencies upon various pathogenic bacteria, including the bacillus typhosus, the vibrio choleræ Asiaticæ, the bacillus pestis bubonicæ, the bacillus dysenteriæ, and the bacillus anthracis. It will be remembered that Dr. Haldane suggested and carried out, also under the direction of the Local Government Board, a series of experiments in rat destruction on shipboard by the agency of carbonic oxide, but the deadly character of this gas, its invisibility, and its freedom from smell, render it an exceedingly dangerous agent for common use, apart from the disadvantage that it has no injurious effect upon the cockroaches and other insects which often swarm in cargo ships, and which may possibly take a share in the conveyance of disease. On all these grounds preparations of sulphur are to be preferred as ordinary disinfectants and lethal agents. They are so pungent as to render their presence manifest, they are practically harmless to mankind, and they may be so applied as to be destructive alike to rodents and to insect vermin. The first part of the report to which we call attention is occupied by Dr. Haldane's account of the application of sulphur to the holds and cargo of the s.s. *Bavaria*, 3,009 tons register, at Dunkirk, by what is known as the Clayton process. The apparatus for this purpose is contained in a barge which is brought alongside of the ship to be treated, and it consists of an iron furnace specially constructed to burn sulphur. Air is drawn through this by means of a Root's blower, the products of combustion being cooled by passing them through a cooling apparatus before they reach the blower. They are driven onwards through a flexible rubber hose which can be introduced into any part of the ship. A return pipe brings air back from the ship to the furnace. The gas produced, of which about 36,000 cubic feet per hour were delivered, consists of the residual nitrogen of the air, with about 15 per cent. of sulphurous acid and a good deal of sulphuric acid in suspension. The mixture is extremely pungent, white, and opaque, and much heavier than air, so that it sinks readily to every part of a closed space into which it is introduced from above.

In dealing with comparatively unoccupied spaces, the results obtained in Dr. Haldane's presence were extremely good. In the men's quarters in the forecastle the proceeding occupied half an hour, after which 10 dead rats and thousands of dead cockroaches were found lying about the floor, while in watching the filling of the saloon with gas rising from below it was observed that all the flies fell dead as the upper level reached them. In the loaded holds the results were less satisfactory, and many live rodents and cockroaches were found after the completion of the operation, while the smell left behind in the bedding and other woollen materials was extremely persistent and unpleasant and was attributed to the use of a sulphur which contained selenium. The cargo, on superficial examination, appeared to be quite uninjured, and the general conclusion was that although the process is most effective in destroying rats and other vermin in cabins and empty holds, there is no satisfactory evidence that it is practicable, without considerable delay and inconvenience, to make it equally effective in holds which are full. The usual treatment of about an hour is declared to be quite ineffective in destroying the rats, mice, and insects, and a series of experiments afterwards instituted appeared to show that the sulphur is almost entirely arrested in, or absorbed by, the superficial portions of bales of compressed goods and never reaches their interior. In these circumstances it would, of course, be inefficacious in the case of packed infected clothing or rags, although it was shown by Dr. Wade that plague bacteria, and probably those of cholera, would be destroyed by a six hours' exposure to a gas containing two per cent. of sulphur dioxide, although the more resistent typhoid microbes would require from twice to four times this time, and although anthrax spores would probably be unaffected in any case. Under circumstances of really effective exposure a good deal of injury was done to some kinds of cargo.

The report concludes with a general summary of the results obtainable by different methods of disinfection or rat destruction, and the authors refer successively to the use of sulphur combustion, of liquid sulphurous acid, of carbonic oxide, and of carboni[illegible] acid. They suggest in conclusion, that it might b[illegible] well to treat the accessible spaces of a ship wit[illegible] sulphurous acid gas before discharge of cargo, [illegible] unload the cargo with all practicable precautions t[illegible] prevent rats from escaping from the holds an[illegible] getting on shore, and then to treat the empty hol[illegible] so effectively with sulphurous acid as to destroy a[illegible] rats and insects. They point out that this cours[illegible] while ostensibly aiming at the ideal in less degr[illegible] than procedures applied before the discharge of cargo, has the important advantages of being practicable at every port, and of being especially suitable in the case of cargoes liable to damage by the acid, and they suggest that it may perhaps be found to give as satisfactory results as more ambitious methods, "not yet free from difficulty in their application nor from doubt as to their efficiency."

